# Vadimezan: 2-(5,6-dimethyl-9-oxo-9*H*-xanthen-4-yl)acetic acid

**DOI:** 10.1107/S1600536810028394

**Published:** 2010-07-21

**Authors:** Shi-Jie Zhang, Wei-Xiao Hu

**Affiliations:** a College of Pharmaceutical Science, Zhejiang University of Technology, Hangzhou 310032, People’s Republic of China

## Abstract

In the title mol­ecule, C_17_H_14_O_4_, the C atom of the carboxyl group deviates by 1.221 (3) Å from the plane [maximum deviation = 0.0122(2) Å] of the tricycic ring system. In the crystal structure, inter­molecular O—H⋯O hydrogen bonds link the mol­ecules into centrosymmetric dimers, and π–π inter­actions [centroid–centroid distances = 3.491 (3), 3.591 (3), 3.639 (3) and 3.735 (3) Å] link these dimers into layers parallel to the *ac* plane. Weak inter­molecular C—H⋯O inter­actions further consolidate the crystal packing.

## Related literature

For general background to and recent reviews of vascular-disrupting agents and the development of Vadimezan (DMXAA, ASA404), a promising small-mol­ecule tumor-vascular disrupting agent in phase III clinical trials, see: McKeage & Baguley (2010 ▶); Head & Jameson (2010 ▶); Ching (2008 ▶); Patterson & Rustin (2007 ▶); Hinnen & Eskens (2007 ▶); Lippert (2007 ▶). For a recent clinical study of Vadimezan, see: Pili *et al.* (2010 ▶); McKeage *et al.* (2008 ▶, 2009 ▶). For studies of the mol­ecular mechanisms and signal pathways of Vadimezan, see: Zhan *et al.* (2010 ▶); Cheng *et al.* (2010 ▶); Roberts *et al.* (2008 ▶). For the biological and pharmacological activity of Vadimezan analogues with structure–activity relationships, see: Gobbi *et al.* (2006 ▶); Woon *et al.* (2005 ▶). For the synthesis and spectroscopic data for Vadimezan, see: Yang & Denny (2009 ▶); Atwell *et al.* (2002 ▶). For related xanthone structures, see: Yu *et al.* (2008 ▶); Zhang *et al.* (2007 ▶).
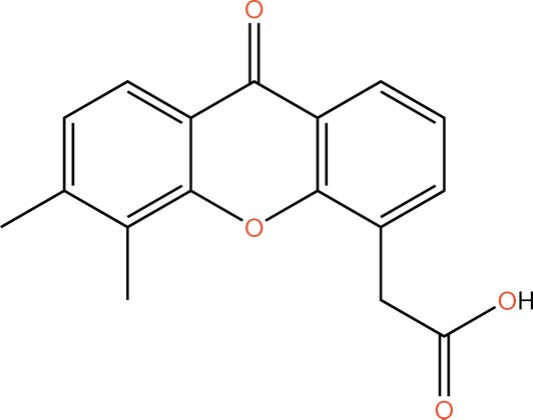


## Experimental

### 

#### Crystal data

C_17_H_14_O_4_
*M* *_r_* = 282.28
Triclinic, 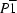

*a* = 6.7854 (19) Å
*b* = 9.826 (3) Å
*c* = 10.532 (3) Å
α = 71.435 (7)°
β = 82.741 (9)°
γ = 83.142 (9)°
*V* = 658.0 (3) Å^3^
*Z* = 2
Mo *K*α radiation
μ = 0.10 mm^−1^
*T* = 163 K
0.50 × 0.50 × 0.37 mm

#### Data collection

Rigaku AFC10/Saturn724+ diffractometer
6284 measured reflections
2955 independent reflections
2304 reflections with *I* > 2σ(*I*)
*R*_int_ = 0.018

#### Refinement

*R*[*F* ^2^ > 2σ(*F* ^2^)] = 0.040
*wR*(*F* ^2^) = 0.094
*S* = 1.00
2955 reflections
195 parameters
H atoms treated by a mixture of independent and constrained refinement
Δρ_max_ = 0.26 e Å^−3^
Δρ_min_ = −0.17 e Å^−3^

### 

Data collection: *CrystalClear* (Rigaku/MSC, 2008 ▶); cell refinement: *CrystalClear*; data reduction: *CrystalClear*; program(s) used to solve structure: *SHELXS97* (Sheldrick, 2008 ▶); program(s) used to refine structure: *SHELXL97* (Sheldrick, 2008 ▶); molecular graphics: *SHELXL97* (Sheldrick, 2008 ▶); software used to prepare material for publication: *publCIF* (Westrip, 2010 ▶) and *PLATON* (Spek, 2009 ▶).

## Supplementary Material

Crystal structure: contains datablocks I, global. DOI: 10.1107/S1600536810028394/cv2747sup1.cif

Structure factors: contains datablocks I. DOI: 10.1107/S1600536810028394/cv2747Isup2.hkl

Additional supplementary materials:  crystallographic information; 3D view; checkCIF report

## Figures and Tables

**Table 1:** Hydrogen-bond geometry (Å, °)

*D*—H⋯*A*	*D*—H	H⋯*A*	*D*⋯*A*	*D*—H⋯*A*
O4—H4*O*⋯O3^iii^	1.00 (2)	1.63 (2)	2.633 (1)	173.25 (2)
C16—H16*B*⋯O4^iv^	0.99	2.52	3.460 (1)	158 (1)

Symmetry codes: (iii) 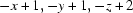; (iv) 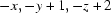.

**Fig. 1.**
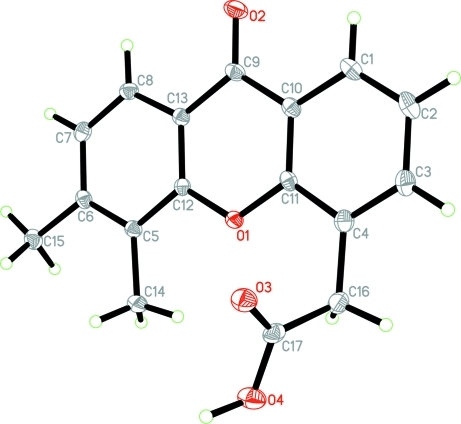
The molecular structure of (I) shown with 30% probability displacement ellipsoids. One component of rotationally disordered methyl group (C14) is shown.

**Fig. 2.**
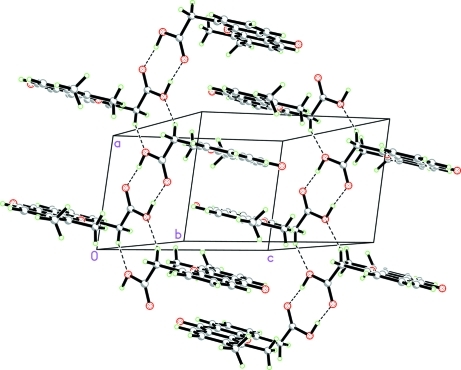
A portion of the packing diagram of (I) showing intermolecular hydrogen bonds as dashed lines.

## supplementary crystallographic information

### Comment

Tumor vascular disrupting agents (VDA) cause the established vascular structure inside a solid tumor to collapse, depriving the tumor of blood, oxygen and nutrients it needs to survive (McKeage *et al.*, 2010; Head *et al.*, 2010; Ching, 2008; Patterson *et al.*, 2007; Hinnen *et al.*, 2007; Lippert, 2007). A number of new drug-based VDAs have been developed that are believed to be highly efficient, low toxic, and several of them are currently undergoing clinical trials, among which Vadimezan is the most advanced in Phase III clinical development (Pili *et al.*, 2010; McKeage *et al.*, 2009; McKeage *et al.*, 2008). The study of antitumor mechanisms and signal pathways of Vadimezan is carried out (Zhan *et al.*, 2010; Cheng *et al.*, 2010; Roberts *et al.*, 2008), as well as the structure modification of xanthones (Gobbi *et al.*, 2006; Woon *et al.*, 2005).

The molecular structure of Vadimezan is very important and necessary in understanding of the compound and may help to further elucidate its antivascular effect for the treatment of human cancer. Its crystal structure is reported for the first time in this paper. The structure (Figure 1) is similar to other xanthones reported with an essential planar three-ring skeleton and the C atom of the carboxyl group deviates at 1.221 (3) Å from the tricycle plane. In the crystal structure, intermolecular O—H···O (Table 2) hydrogen bonds link the molecules into centrosymmetric dimers, and π-π interactions (Table 1) link these dimers into layers parallel to *ac* plane. Weak intermolecular C—H···O interactions (Table 2) consolidate further the crystal packing (Fig. 2).

### Experimental

Vadimezan was prepared from 3,4-dimethylanthranilic acid according to literature method (Atwell *et al.*, 2002). Diazotization of 3,4-dimethylanthranilic acid with NaNO_2_ and then treatment with KI led to 3,4-dimethyl-2-iodobenzoic acid, which was coupled with 2-hydroxyphenylacetic acid catalyzed by TDA-1 and Cu^(I)^ in dried DMSO to obtain 2-[2-(carboxylmethyl)phenoxy]-3,4-dimethylbenzoic acid. Finally, ring closure in sulfuric acid led to 5,6-dimethyl-9-oxoxanthene-4-acetic acid: white solid; mp: 529–532 K; *P*_HPLC_ 98.2%; IR ν_max_ (KBr)/cm^-1^: 2970, 1708, 1650, 1602, 1411, 1330, 1212, 768; ^1^H NMR (DMSO-d_6_, 500 MHz) δ: 12.60 (s, 1H, COOH), 8.07 (dd, *J_1_* = 1.5 Hz, *J_2_* = 8.0 Hz, 1H, Ar), 7.89 (d, *J* = 8.0 Hz, 1H, Ar), 7.78 (dd, *J_1_* = 1.3 Hz, *J_2_* = 7.3 Hz, 1H, Ar), 7.40 (t, *J* = 7.5 Hz, 1H, Ar), 7.24 (d, *J* = 8.5 Hz, 1H, Ar), 3.95 (s, 2H, Ar—CH_2_), 2.39 (s, 3H, Ar—CH_3_), 2.36 (s, 3H, Ar—CH_3_); EIMS m/*z*(%): 282(*M*^+^, 77), 238 (42), 237 (100), 236 (26), 223 (17), 209 (37), 195 (12), 165 (28).

### Refinement

H atom attached to carboxyl O atom was located in a difference map and refined with bond restraint O—H = 1.00 (2) Å. C-bound H atoms were positioned geometrically (C—H 0.95 - 0.99 Å). All H atoms were refined as riding, with *U*_iso_(H) = 1.2 - 1.5 *U*_eq_ of the parent atoms. Hydrogen atoms attached to C14 are disordered with a refined site occupancy factor 0.50 for each H14A, H14B, H14C, H14D, H14E and H14F.

### Crystal data

**Table:**

C_17_H_14_O_4_	*Z* = 2
*M**_r_* = 282.28	*F*(000) = 296
Triclinic, *P*1	*D*_x_ = 1.425 Mg m^−^^3^
*a* = 6.7854 (19) Å	Mo *K*α radiation, λ = 0.71073 Å
*b* = 9.826 (3) Å	Cell parameters from 1944 reflections
*c* = 10.532 (3) Å	θ = 3.0–27.5°
α = 71.435 (7)°	µ = 0.10 mm^−^^1^
β = 82.741 (9)°	*T* = 163 K
γ = 83.142 (9)°	Block, colorless
*V* = 658.0 (3) Å^3^	0.50 × 0.50 × 0.37 mm

### Data collection

**Table:**

Rigaku AFC10/Saturn724+ diffractometer	2304 reflections with *I* > 2σ(*I*)
Radiation source: Rotating Anode	*R*_int_ = 0.018
graphite	θ_max_ = 27.5°, θ_min_ = 3.4°
Detector resolution: 28.5714 pixels mm^-1^	*h* = −8→8
phi and ω scans	*k* = −11→12
6284 measured reflections	*l* = −13→13
2955 independent reflections	

### Refinement

**Table:**

Refinement on *F*^2^	Primary atom site location: structure-invariant direct methods
Least-squares matrix: full	Secondary atom site location: difference Fourier map
*R*[*F*^2^ > 2σ(*F*^2^)] = 0.040	Hydrogen site location: inferred from neighbouring sites
*wR*(*F*^2^) = 0.094	H atoms treated by a mixture of independent and constrained refinement
*S* = 1.00	*w* = 1/[σ^2^(*F*_o_^2^) + (0.0346*P*)^2^ + 0.226*P*] where *P* = (*F*_o_^2^ + 2*F*_c_^2^)/3
2955 reflections	(Δ/σ)_max_ < 0.001
195 parameters	Δρ_max_ = 0.26 e Å^−^^3^
0 restraints	Δρ_min_ = −0.17 e Å^−^^3^

### Special details

**Table:**

Geometry. All e.s.d.'s (except the e.s.d. in the dihedral angle between two l.s. planes) are estimated using the full covariance matrix. The cell e.s.d.'s are taken into account individually in the estimation of e.s.d.'s in distances, angles and torsion angles; correlations between e.s.d.'s in cell parameters are only used when they are defined by crystal symmetry. An approximate (isotropic) treatment of cell e.s.d.'s is used for estimating e.s.d.'s involving l.s. planes.
Refinement. Refinement of *F*^2^ against ALL reflections. The weighted *R*-factor *wR* and goodness of fit *S* are based on *F*^2^, conventional *R*-factors *R* are based on *F*, with *F* set to zero for negative *F*^2^. The threshold expression of *F*^2^ > σ(*F*^2^) is used only for calculating *R*-factors(gt) *etc*. and is not relevant to the choice of reflections for refinement. *R*-factors based on *F*^2^ are statistically about twice as large as those based on *F*, and *R*- factors based on ALL data will be even larger.

### Fractional atomic coordinates and isotropic or equivalent isotropic displacement parameters (Å^2^)

**Table:**

	*x*	*y*	*z*	*U*_iso_*/*U*_eq_	Occ. (<1)
O1	0.21097 (13)	0.40487 (9)	0.72385 (9)	0.0215 (2)	
O2	0.36821 (16)	0.20951 (10)	0.43040 (10)	0.0335 (3)	
O3	0.49314 (15)	0.35197 (11)	0.95899 (10)	0.0312 (2)	
O4	0.23835 (16)	0.47712 (12)	1.03729 (11)	0.0361 (3)	
C1	0.3402 (2)	0.03641 (14)	0.70661 (15)	0.0278 (3)	
H1	0.3796	−0.0144	0.6431	0.033*	
C2	0.3195 (2)	−0.03755 (15)	0.84129 (16)	0.0330 (3)	
H2	0.3434	−0.1395	0.8710	0.040*	
C3	0.2632 (2)	0.03676 (15)	0.93484 (15)	0.0306 (3)	
H3	0.2494	−0.0159	1.0279	0.037*	
C4	0.2271 (2)	0.18497 (14)	0.89551 (14)	0.0252 (3)	
C5	0.18653 (18)	0.63568 (13)	0.56645 (13)	0.0208 (3)	
C6	0.20182 (18)	0.72389 (13)	0.43318 (13)	0.0221 (3)	
C7	0.25597 (19)	0.66243 (14)	0.32918 (13)	0.0248 (3)	
H7	0.2664	0.7233	0.2386	0.030*	
C8	0.29413 (19)	0.51639 (14)	0.35551 (13)	0.0238 (3)	
H8	0.3301	0.4772	0.2833	0.029*	
C9	0.32191 (19)	0.26831 (14)	0.51829 (13)	0.0232 (3)	
C10	0.30347 (19)	0.18691 (14)	0.66213 (13)	0.0223 (3)	
C11	0.24803 (18)	0.25830 (13)	0.75729 (13)	0.0212 (3)	
C12	0.22678 (17)	0.48664 (13)	0.59068 (12)	0.0188 (3)	
C13	0.28044 (18)	0.42500 (13)	0.48780 (13)	0.0202 (3)	
C14	0.1303 (2)	0.69521 (15)	0.68242 (14)	0.0298 (3)	
H14A	0.1279	0.6159	0.7671	0.036*	0.50
H14B	−0.0020	0.7477	0.6734	0.036*	0.50
H14C	0.2284	0.7608	0.6823	0.036*	0.50
H14D	0.1084	0.8004	0.6481	0.036*	0.50
H14E	0.2382	0.6686	0.7418	0.036*	0.50
H14F	0.0078	0.6554	0.7330	0.036*	0.50
C15	0.1628 (2)	0.88493 (14)	0.39994 (15)	0.0291 (3)	
H15A	0.0275	0.9087	0.4362	0.035*	
H15B	0.1761	0.9292	0.3021	0.035*	
H15C	0.2594	0.9217	0.4399	0.035*	
C16	0.1661 (2)	0.26804 (15)	0.99384 (14)	0.0293 (3)	
H16A	0.1462	0.1991	1.0854	0.035*	
H16B	0.0369	0.3240	0.9720	0.035*	
C17	0.3156 (2)	0.36947 (15)	0.99384 (13)	0.0260 (3)	
H4O	0.345 (3)	0.541 (2)	1.032 (2)	0.076 (7)*	

### Atomic displacement parameters (Å^2^)

**Table:**

	*U*^11^	*U*^22^	*U*^33^	*U*^12^	*U*^13^	*U*^23^
O1	0.0261 (5)	0.0176 (4)	0.0205 (5)	−0.0008 (3)	−0.0016 (4)	−0.0061 (4)
O2	0.0449 (6)	0.0277 (5)	0.0326 (6)	0.0000 (4)	−0.0035 (5)	−0.0171 (4)
O3	0.0298 (5)	0.0314 (5)	0.0339 (6)	0.0002 (4)	−0.0023 (4)	−0.0133 (4)
O4	0.0322 (6)	0.0397 (6)	0.0430 (6)	0.0003 (5)	−0.0014 (5)	−0.0241 (5)
C1	0.0279 (7)	0.0212 (7)	0.0377 (8)	−0.0008 (5)	−0.0081 (6)	−0.0125 (6)
C2	0.0367 (8)	0.0185 (7)	0.0426 (9)	−0.0008 (6)	−0.0115 (7)	−0.0053 (6)
C3	0.0334 (8)	0.0252 (7)	0.0295 (7)	−0.0046 (6)	−0.0071 (6)	−0.0010 (6)
C4	0.0227 (7)	0.0253 (7)	0.0272 (7)	−0.0041 (5)	−0.0031 (5)	−0.0063 (6)
C5	0.0184 (6)	0.0207 (6)	0.0249 (7)	−0.0018 (5)	−0.0017 (5)	−0.0094 (5)
C6	0.0185 (6)	0.0198 (6)	0.0279 (7)	−0.0021 (5)	−0.0036 (5)	−0.0063 (5)
C7	0.0239 (7)	0.0266 (7)	0.0215 (7)	−0.0030 (5)	−0.0030 (5)	−0.0034 (5)
C8	0.0233 (7)	0.0278 (7)	0.0226 (7)	−0.0029 (5)	−0.0016 (5)	−0.0108 (5)
C9	0.0211 (6)	0.0232 (7)	0.0290 (7)	−0.0019 (5)	−0.0038 (5)	−0.0127 (6)
C10	0.0197 (6)	0.0206 (6)	0.0287 (7)	−0.0021 (5)	−0.0052 (5)	−0.0089 (5)
C11	0.0185 (6)	0.0179 (6)	0.0273 (7)	−0.0024 (5)	−0.0042 (5)	−0.0060 (5)
C12	0.0159 (6)	0.0199 (6)	0.0206 (6)	−0.0030 (5)	−0.0019 (5)	−0.0058 (5)
C13	0.0175 (6)	0.0209 (6)	0.0241 (7)	−0.0025 (5)	−0.0030 (5)	−0.0089 (5)
C14	0.0385 (8)	0.0228 (7)	0.0289 (7)	−0.0018 (6)	0.0008 (6)	−0.0108 (6)
C15	0.0318 (8)	0.0218 (7)	0.0317 (8)	−0.0016 (5)	−0.0032 (6)	−0.0053 (6)
C16	0.0307 (7)	0.0295 (7)	0.0246 (7)	−0.0036 (6)	0.0015 (6)	−0.0053 (6)
C17	0.0320 (7)	0.0270 (7)	0.0165 (6)	0.0022 (6)	−0.0036 (5)	−0.0045 (5)

### Geometric parameters (Å, °)

**Table:**

O1—C11	1.3690 (15)	C7—C8	1.3708 (19)
O1—C12	1.3746 (15)	C7—H7	0.9500
O2—C9	1.2301 (15)	C8—C13	1.3953 (18)
O3—C17	1.2225 (17)	C8—H8	0.9500
O4—C17	1.3108 (16)	C9—C10	1.4680 (19)
O4—H4O	1.00 (2)	C9—C13	1.4688 (18)
C1—C2	1.370 (2)	C10—C11	1.3883 (18)
C1—C10	1.4032 (18)	C12—C13	1.3925 (17)
C1—H1	0.9500	C14—H14A	0.9800
C2—C3	1.395 (2)	C14—H14B	0.9800
C2—H2	0.9500	C14—H14C	0.9800
C3—C4	1.3816 (19)	C14—H14D	0.9800
C3—H3	0.9500	C14—H14E	0.9800
C4—C11	1.4011 (19)	C14—H14F	0.9800
C4—C16	1.5030 (19)	C15—H15A	0.9800
C5—C6	1.3937 (18)	C15—H15B	0.9800
C5—C12	1.4031 (17)	C15—H15C	0.9800
C5—C14	1.5045 (18)	C16—C17	1.505 (2)
C6—C7	1.4021 (19)	C16—H16A	0.9900
C6—C15	1.5057 (18)	C16—H16B	0.9900
			
Cg1···Cg2^i^	3.491 (3)	Cg2···Cg2^i^	3.735 (3)
Cg1···Cg2^ii^	3.591 (3)	Cg2···Cg2^ii^	3.639 (3)
			
C11—O1—C12	119.45 (10)	C12—C13—C9	120.73 (12)
C17—O4—H4O	109.3 (12)	C8—C13—C9	121.21 (11)
C2—C1—C10	120.21 (13)	C5—C14—H14A	109.5
C2—C1—H1	119.9	C5—C14—H14B	109.5
C10—C1—H1	119.9	H14A—C14—H14B	109.5
C1—C2—C3	120.06 (13)	C5—C14—H14C	109.5
C1—C2—H2	120.0	H14A—C14—H14C	109.5
C3—C2—H2	120.0	H14B—C14—H14C	109.5
C4—C3—C2	121.64 (13)	C5—C14—H14D	109.5
C4—C3—H3	119.2	H14A—C14—H14D	141.1
C2—C3—H3	119.2	H14B—C14—H14D	56.3
C3—C4—C11	117.33 (12)	H14C—C14—H14D	56.3
C3—C4—C16	122.88 (13)	C5—C14—H14E	109.5
C11—C4—C16	119.79 (12)	H14A—C14—H14E	56.3
C6—C5—C12	117.71 (11)	H14B—C14—H14E	141.1
C6—C5—C14	122.29 (11)	H14C—C14—H14E	56.3
C12—C5—C14	120.00 (12)	H14D—C14—H14E	109.5
C5—C6—C7	119.79 (12)	C5—C14—H14F	109.5
C5—C6—C15	120.53 (11)	H14A—C14—H14F	56.3
C7—C6—C15	119.67 (12)	H14B—C14—H14F	56.3
C8—C7—C6	121.37 (12)	H14C—C14—H14F	141.1
C8—C7—H7	119.3	H14D—C14—H14F	109.5
C6—C7—H7	119.3	H14E—C14—H14F	109.5
C7—C8—C13	120.32 (12)	C6—C15—H15A	109.5
C7—C8—H8	119.8	C6—C15—H15B	109.5
C13—C8—H8	119.8	H15A—C15—H15B	109.5
O2—C9—C10	122.46 (12)	C6—C15—H15C	109.5
O2—C9—C13	122.77 (12)	H15A—C15—H15C	109.5
C10—C9—C13	114.77 (11)	H15B—C15—H15C	109.5
C11—C10—C1	118.59 (12)	C4—C16—C17	113.59 (11)
C11—C10—C9	120.20 (11)	C4—C16—H16A	108.8
C1—C10—C9	121.21 (12)	C17—C16—H16A	108.8
O1—C11—C10	122.88 (12)	C4—C16—H16B	108.8
O1—C11—C4	114.95 (11)	C17—C16—H16B	108.8
C10—C11—C4	122.17 (12)	H16A—C16—H16B	107.7
O1—C12—C13	121.97 (11)	O3—C17—O4	123.02 (14)
O1—C12—C5	115.28 (11)	O3—C17—C16	123.28 (12)
C13—C12—C5	122.75 (12)	O4—C17—C16	113.68 (12)
C12—C13—C8	118.06 (11)		
			
C10—C1—C2—C3	−0.5 (2)	C16—C4—C11—O1	0.55 (18)
C1—C2—C3—C4	0.0 (2)	C3—C4—C11—C10	−0.25 (19)
C2—C3—C4—C11	0.4 (2)	C16—C4—C11—C10	−179.84 (12)
C2—C3—C4—C16	179.95 (13)	C11—O1—C12—C13	−0.27 (17)
C12—C5—C6—C7	−0.03 (18)	C11—O1—C12—C5	−179.83 (11)
C14—C5—C6—C7	179.62 (12)	C6—C5—C12—O1	179.53 (11)
C12—C5—C6—C15	−179.37 (12)	C14—C5—C12—O1	−0.13 (17)
C14—C5—C6—C15	0.28 (19)	C6—C5—C12—C13	−0.03 (19)
C5—C6—C7—C8	0.2 (2)	C14—C5—C12—C13	−179.69 (12)
C15—C6—C7—C8	179.51 (12)	O1—C12—C13—C8	−179.58 (11)
C6—C7—C8—C13	−0.2 (2)	C5—C12—C13—C8	−0.05 (19)
C2—C1—C10—C11	0.6 (2)	O1—C12—C13—C9	0.23 (18)
C2—C1—C10—C9	−179.11 (13)	C5—C12—C13—C9	179.75 (11)
O2—C9—C10—C11	−179.09 (12)	C7—C8—C13—C12	0.19 (19)
C13—C9—C10—C11	0.80 (17)	C7—C8—C13—C9	−179.61 (12)
O2—C9—C10—C1	0.6 (2)	O2—C9—C13—C12	179.41 (12)
C13—C9—C10—C1	−179.46 (12)	C10—C9—C13—C12	−0.47 (17)
C12—O1—C11—C10	0.62 (17)	O2—C9—C13—C8	−0.8 (2)
C12—O1—C11—C4	−179.77 (11)	C10—C9—C13—C8	179.32 (11)
C1—C10—C11—O1	179.34 (12)	C3—C4—C16—C17	116.51 (15)
C9—C10—C11—O1	−0.91 (19)	C11—C4—C16—C17	−63.93 (17)
C1—C10—C11—C4	−0.24 (19)	C4—C16—C17—O3	−25.38 (19)
C9—C10—C11—C4	179.51 (12)	C4—C16—C17—O4	155.89 (12)
C3—C4—C11—O1	−179.87 (11)		

Symmetry codes: (i) −*x*+1, −*y*+1, −*z*+1; (ii) −*x*+2, −*y*+1, −*z*+1.

### Hydrogen-bond geometry (Å, °)

**Table:**

*D*—H···*A*	*D*—H	H···*A*	*D*···*A*	*D*—H···*A*
O4—H4O···O3^iii^	1.00 (2)	1.63 (2)	2.633 (1)	173.25 (2)
C16—H16B···O4^iv^	0.99	2.52	3.460 (1)	158 (1)

Symmetry codes: (iii) −*x*+1, −*y*+1, −*z*+2; (iv) −*x*, −*y*+1, −*z*+2.
